# Climate Change and Mental Health: A Review of Empirical Evidence, Mechanisms and Implications

**DOI:** 10.3390/atmos13122096

**Published:** 2022-12-13

**Authors:** Katelin Crane, Linda Li, Pearl Subramanian, Elizabeth Rovit, Jianghong Liu

**Affiliations:** 1 School of Nursing, University of Pennsylvania, Philadelphia, PA 19104, USA; 2 Department of Anesthesiology and Perioperative Medicine, University of California, Los Angeles, CA 90095, USA; 3 Donald and Barbara Zucker School of Medicine, Hempstead, NY 11549, USA; 4 Lewis Katz School of Medicine, Temple University, Philadelphia, PA 19140, USA

**Keywords:** climate change, mental health, global warming, suicide, PTSD, depression

## Abstract

Anthropogenic climate change is an existential threat whose influences continue to increase in severity. It is pivotal to understand the implications of climate change and their effects on mental health. This integrative review aims to summarize the relevant evidence examining the harm climate change may have on mental health, suggest potential mechanisms and discuss implications. Empirical evidence has begun to indicate that negative mental health outcomes are a relevant and notable consequence of climate change. Specifically, these negative outcomes range from increased rates of psychiatric diagnoses such as depression, anxiety and post-traumatic stress disorder to higher measures of suicide, aggression and crime. Potential mechanisms are thought to include neuroinflammatory responses to stress, maladaptive serotonergic receptors and detrimental effects on one’s own physical health, as well as the community wellbeing. While climate change and mental health are salient areas of research, the evidence examining an association is limited. Therefore, further work should be conducted to delineate exact pathways of action to explain the mediators and mechanisms of the interaction between climate change and mental health.

## 1. Introduction

The widespread consequences of human-caused global warming are indisputable. While once considered a distant threat, the damage climate change has already wreaked on the planet has and will continue to transform vital aspects of our world. While climate change undoubtedly poses significant impacts to physical health, as nearly 12.6 million preventable deaths per year are attributed to environmental changes [1], recent literature indicates that anthropogenic climate change negatively affects mental health as well. It has been estimated that, in individuals who experience extreme weather phenomena, a direct impact of climate change, between 25% and 50% will develop harmful mental health symptoms that impact their quality of life [2].

These impacts appear to be multifaceted and complex. A common initial response to experiencing a traumatic event, including a climate change-related disaster (hurricanes, wildfires and tornadoes) appear to be parallel with the symptoms of a trauma response–avoidance, guilt, rumination, hypervigilance and nightmares, among others [2]. Research shows that many of these symptoms improve over time, but a significant number of individuals develop diagnosable mental health disorders [3].

Current literature views climate change and its effects through one of three lenses: as an outcome of acute climate-related disasters such as floods, hurricanes, wildfires, or tornadoes; subacute climate change incidents which are often slow-progressing and less visible and lead to gradual changes such as increased temperatures, drought and air pollution; or the potential prolonged effects of acute and subacute climate change-related disasters and their chronic impact on community health [2]. It is suspected that the environmental changes related to climate change can result in economic losses, widely discussed threats to physical health, individuals being displaced from their homes due to property damage, social conflict and inter-group violence, all of which can remarkably alter mental health. For example, data suggests that mental health conditions such as post-traumatic stress disorder (PTSD) and suicide/suicidal thoughts resulting from weather extremes and natural disasters may stem from witnessing the severe injury of death of family or friends or forced displacement from one’s home [4]. Additionally, other climate change-related disasters such as heatwaves may lead to the exacerbation of schizophrenia and psychosis and exacerbations in acute and chronic mental health disorders can be witnessed in both elderly, children and young adults alike [5,6]. The emerging evidence of damaging effects of climate change on mental health underscores the necessity for further investigation.

As global temperatures, sea levels and carbon dioxide levels continue to rise, a deeper understanding of the effects of climate change on mental health has never been more pertinent for policy and public health efforts. The aim of this paper is thus to review the recent empirical evidence regarding effects of climate change, as defined by acute, subacute and chronic exposures and their associated mental health outcomes. We hypothesize that acute, subacute and chronic events secondary to anthropogenic climate change are associated with negative consequences on mental health. Through this literature review, we hope to emphasize the need for policy and preventative solutions surrounding climate change, so that both physical and mental health are considered in emergency preparedness planning.

### Definition of Climate Change

Climate change refers to a long-term significant change in the average patterns of weather across Earth’s local, regional and global climates. While natural processes such as El Niño, La Niña, volcanic activity and variations in Earth’s orbit can contribute to climate change, most changes occurring since the early 20th century are predominantly caused by human behavior [7]. A key indicator of the overall health of Earth’s climate, the global surface temperature, is pivotal in understanding climate change. The Sixth Assessment Report conducted by the Intergovernmental Panel on Climate Change found that the globe is warming and it is warming rapidly. While there have been fluctuations between relatively warm and glacial over the past two million years, the shift from the last glacial period to the current interglacial period has produced an overall temperature increase of about 5 °C, which took place over the span of about 5000 years, with the maximum yearly increase in temperature of about 1.5 °C per thousand years [8]. In jarring contrast, Earth has warmed by approximately 1.1 °C since 1850–1900—the last time it was this warm on earth was the previous interglacial period, which took place around 125,000 years ago [8].

Humans—or rather the greenhouse gases we produce—are the leading cause of climate change today [9]. Environmental scientists have linked the root of greenhouse gas emissions to humans by collecting carbon isotope fingerprints. Fossil fuels produce ratios of stable carbon-13 to carbon-12 that differs from the stable carbon ratio in Earth’s atmosphere [10]. Compared to signatures collected from Antarctic Ice Sheets that show stable carbon isotopic signatures from ~1000 AD to ~1800 AD followed by a steady change in isotopic signature since 1800 and rapid change since 1950, carbon isotopic signatures from fossil fuels are increasing as a percentage [10].

In the early 2000s, climate activism, which has roots going back at least to the late 1960s, became increasingly mainstream. With growing evidence to support the magnitude and danger of climate change, this movement eventually led to the implementation of the Paris Agreement in 2015, in which over 160 countries joined together to commit to reducing greenhouse gas emissions [11]. While the Paris Agreement concerns multitudinous issues, it has helped to bring to the forefront the stark realities of climate change. The countries in the agreement put forth a commitment to help reduce fossil fuel combustion. Furthermore, in 2021, a bill titled H.R. 794-Climate Emergency Act of 2021 was introduced in the United States House of Representatives that would ensure that the President of the United States would necessitate that, amongst other things, the government “invests in large scale mitigation and resiliency projects; makes investments that enable a racially and socially just transition to a clean energy economy by ensuring that at least 40% of investments flow to historically disadvantaged communities; combats environmental injustice; and reinvests in existing public sector institutions and creates new public sector institutions to strategically mobilize and channel investments at the scale and pace required by the national emergency” [12]. With escalating severity, the negative effects previously discussed have led to devastating and costly consequences. While climate changes affect us all, it is irrefutable that disadvantaged groups, such as women, people of color, indigenous communities and children will be disproportionately impacted. As such, climate change is a human rights issue that needs to be immediately addressed.

## 2. Materials and Methods

An integrative review of the literature was performed using parameters outlined by Russell [13] with the purpose of summarizing and synthesizing current literature on the multitude of effects on climate change and mental health. We identified the following research question: how might mental health be impacted by climate change and what are the mechanisms by which this happens? A search strategy was created that involved terms related to climate change, such as ‘global warming’, ‘natural disaster’, ‘and ‘climate’, as well as terms related to potential impacts on mental health, such as ‘depression’, ‘PTSD’, ‘suicide’ and ‘violence’. Three databases (PsycINFO, PubMed and SCOPUS) were searched. In order to be deemed eligible, studies needed to present primary data, be published in English in peer-reviewed journals, have a high impact factor or be well-cited. While we focused our search on literature that was published in the last five years, we also included studies published earlier if highly relevant and well-cited. We excluded studies that were not well-cited or had a low impact factor. Conference papers, dissertations, letters and opinion pieces were also excluded. Thematic analysis was used to explore patterns that emerged in the literature.

## 3. Results

### 3.1. Current Evidence of Climate Change and Mental Health

Many studies that examine the role of climate change on mental health underscore the threat of harm to both individuals and communities. However, there are methodological limitations, as studies examining outcomes after severe weather events are mainly cross-sectional, with few studies that include longitudinal pre-disaster data. On the contrary, it is common for studies examining chronic effects, such as rising temperatures, to draw conclusions that are correlative rather than causative. Many studies have endorsed a broader definition of psychological impacts and will utilize self- or parent-reported questionnaires that screen for symptoms of internalizing and/or externalizing behaviors rather than the formal diagnosis of a clinical disorder [14,15]. Furthermore, studies have also looked at outcomes such as emergency department visits and violent crime as proxies for mental health problems [16,17]. This is not to say that these studies do not have value; however, it is also important to further examine the direct impacts of climate change on mental health. Table 1 outlines recent evidence on the potential impact of anthropogenic climate change on mental health. Studies are categorized by type of climate change-related event. Not all studies listed in Table 1 are cited in the main text for page length and clarity.

### 3.2. Risk Factors

In order to create more targeted public health interventions, it is integral to recognize risk factors and identify populations that may be particularly vulnerable to the effects of climate change. While individuals that live through a climate disaster may all experience psychological and sociological distress, it is inaccurate to assume that all of them will go on to develop a diagnosable mental health disorder. In fact, many symptoms of the immediate responses to the traumatic event tend to resolve over time and most can fully recover after a disaster [3]; however, some who develop symptoms after a traumatic event will develop a diagnosable mental health disorder [18]. There are many risk factors for developing a mental illness in the aftermath of a climate-related disaster, including the severity of the disaster, being a witness (firsthand or secondhand) of death or injury, female gender, lower education or socioeconomic status, younger age, minority or ethnic status, psychiatric history, family instability and inadequate social support [3,17]. Contrarily, there are also several determinants of resilience that have the potential to increase adaptability following a weather-related disaster. Those who possess self-efficacy regarding coping is an important predictor—those who believe they can cope with whatever comes their way fare better than those that do not [19]. Yet overcoming psychological adversity depends on much more than just will and personal resources. People that are ingratiated into a larger community are significantly more likely to recover if the community is socially supportive—it is hypothesized that a lack of social support can compound mental anguish [20].

### 3.3. Impacts of Acute, Subacute and Chronic Climate Change-Related Events

Acute, subacute and chronic consequences of climate change are all associated with negative mental health outcomes. While the mechanisms of action that climate change-related events have on the psychopathology of affected individuals remains unknown, several hypotheses have been proposed, such as a pre-existing lower level of resiliency to natural disasters, or the weather event’s magnification of additional psychosocial stressors [4].

There is a substantial body of evidence that has documented the mental health outcomes after acute, short-term (i.e., lasting for days) extreme weather events such as hurricanes, floods, wildfires and heatwaves. Negative sequelae that are commonly associated with acute consequences of climate change include heightened anxiety, acute stress reactions, sleep disruption and a decreased sense of self and self-identity due to displacement from homes and communities [21]. A recent event that exemplifies the intersection of extreme weather events and their potential to wreak havoc on pre-existing vulnerabilities is Hurricane Maria that made landfall on Puerto Rico in October 2017. At that time, Puerto Rico was in the throes of a decade-long recession that had led to skyrocketing rates of unemployment, poverty and separated families due to emigration. In the nine months following the hurricane, acute mental health concerns were greatly exacerbated—suicides rose by a staggering 18% and calls to Puerto Rico’s main suicide hotline rose by 13% when compared to the previous year [22].

Subacute or long-term climate-related changes last for months or years and include events such as drought and long-duration heat waves. These impacts may provoke an intense emotional response in those who experience the effects of climate change both directly and indirectly and lead to anxiety related to an uncertainty about the future of humans, other species and the planet [23]. In a large-scale longitudinal study conducted on two million randomly sampled US residents between 2002 and 2012, Obradovich et al. found that an increase in monthly temperatures between 25 °C and 30 °C to more than 30° increased the likelihood of negative mental health events by 0.5% and that a 1 °C temperature increase over five years is associated with a 2% increase of negative mental health events [24]. Similarly, another study found that suicide rates in the United States and Mexico rose by 0.7% and 3.1%, respectively, for every 1 °C increase in monthly temperature—which will result in a combined 21,770 (95% CI 8950–39,260) additional suicides by 2050 [25]. Additionally, heat suppresses thyroid hormones which results in functional hypothyroidism, which can present as decreased energy, dysphoria and cognitive impairment [26]. Drought presents its own set of outcomes that cast an interesting light on the indirect impacts of climate-related events. One review discovered an economic effects pathway that has particularly severe impacts on rural farming populations, as well as a migration pathway that links drought to mental health [27]. Additionally, a causal-pathways model suggested two indirect mechanisms by which subacute climate change-related events are thought to impact mental health: (1) by negatively impacting physical health through increased heat stress, disease and disruption to food supply and supply chain; and (2) by deteriorating community wellbeing through damage to the economic and social fabric of communities [28].

Even if planned mitigation efforts to combat global climate change are successful, a significant amount of damage has already occurred to many areas of the world. While not all regions are experiencing acute or subacute weather events such as the ones described above, we all are affected by the long-lasting climate-related events such as economic deficits, global conflicts and environmental degradation. It is important to note that poor countries will continue to be the most adversely affected due to the greater reliance on farming and exposure to higher overall temperatures, which will place even more stress on already limited access to infrastructure and risk management [29]. Perhaps the most profound mental health outcome related to long-lasting climate-related events, however, is the psychological threat to the human existence that comes from the conceptual realization of anthropogenic climate change [30,31]. Indeed, “(t)his awareness contributes to ‘psycho-terratic’ syndromes, including phenomena such as ‘ecoanxiety’, ‘eco-paralysis’ and ‘solastalgia’, the distress and isolation caused by the gradual removal of solace from the present state of one’s home environment” [21]. Research has demonstrated that younger generations, particularly those living in wealthier countries, are believed to be particularly impacted by these syndromes [32].

### 3.4. Internalizing and Externalizing Mental Health Behaviors

As outlined above, acute, subacute and chronic consequences of climate change are associated with a variety of negative sequelae, such as increased psychological distress, mental health-related emergency room visits and admissions, suicide, depression, anxiety and development of disorders such as post-traumatic stress disorder (PTSD). As outlined in Table 1, the literature focuses primarily on identifying the course of mental health changes following extreme weather-related events, which may result in conditions such as PTSD, depression, anxiety and suicidal behavior. Internalizing behaviors are negative behaviors that focus inwardly towards the self, such as depression, anxiety, social withdrawal and suicide, whereas externalizing behaviors are negative behaviors that are directed outward toward others such as aggression, opposition, defiance and violence [33].

### 3.5. Internalizing Mental Health Behaviors

#### 3.5.1. PTSD

Acute stress reactions, adjustment disorders and post-traumatic stress disorder (PTSD) are commonly experienced mental health conditions following climate change-related disasters. Such events have the potential to disrupt life and cause catastrophic losses, which may lead to psychiatric responses such as hypervigilance, guilt, fear, or flashbacks [2]. In a sense, these experiences could be considered a normal response to an abnormal situation. However, when prolonged, trauma-based symptomatology following a climate change-related disaster has the potential to lead to diagnosable PTSD. The rates of PTSD post-disaster are staggering—estimates range between 25% to 40% and can result from nearly every type of weather disaster, including wildfires, hurricanes and earthquakes, without regard to continent or culture [2,21,59]. Disaster-related PTSD does not impact survivors equally. There is a body of research that indicates that incidence of post-disaster PTSD is higher among females compared to males, older people compared to younger people, those of low socioeconomic status or the unemployed, or those that have pre-existing mental health disorders [60].

The extent of exposure to and experience of a natural disaster appears to yield a dose-dependent relationship with the development of PTSD symptoms. After the Victorian Black Saturday bushfires, probable PTSD was identified in 15.6% of subjects living in highly-affected areas and significantly more than the 7.2% and 1% of subjects in medium- and low-affected areas, respectively (*p* < 0.001) [37]. Similarly, in an area of extensive flooding in Australia, adults who were personally affected by floods scored significantly higher PTSD scores than those not personally affected (*p* = 0.001) [14]. Disaster-related PTSD may persist for several years after the initial disaster [38] and may stem from disaster-related experiences including serious injury or death of a someone close, fear for one’s life, or displacement from home [37,38,42,43]. In a study of 130 adults affected by Hurricane Sandy, Schwartz and colleagues examined mental health outcomes (depression, anxiety, PTSD) in relation to several personal (e.g., directly affecting participant or family) and property exposures (e.g., level of property affected, financial hardship) [43]. At follow-up (1 year after initial evaluation post-disaster), PTSD was significantly associated with experiencing personal damage (OR 1.6, 95% CI [1.2–2.2]), property damage (OR 1.3, 95% CI [1.1–1.5]), or both (OR 1.2, 95% CI [1.1–1.4]); however, there were no statistically significant increased odds of these exposures to depression or anxiety, suggesting that the impacts of disaster may uniquely have persisting effects on PTSD.

##### Proposed Mechanisms of Climate Change-Related PTSD

The specific mechanism of action in which climate change leads to developing PTSD remains unknown; however, it is hypothesized that multiple brain systems may be implicated, both at the structural and chemical level. Studies have shown that patients with PTSD had decreased volume in several regions of the brain, including the hippocampus, left amygdala and anterior cingulate cortex, all thought to play a role in memory processing and emotion regulation [61,62]. Neurotransmitter changes in the adrenergic receptor system result in increased norepinephrine levels and downregulated adrenergic receptors, as well as decreased glucocorticoid levels and upregulated glucocorticoid receptors [63]. These changes in the anatomical and neurochemical pathways are believed to drive the core symptoms of the disorder.

#### 3.5.2. Depression

Compared to acute stress responses, which tend to be relatively short-lasting after surviving extreme weather events, depressive symptoms and diagnosable major depressive disorder emerge as a longer-standing effect. Like PTSD, there is a breadth of literature that correlates mood disorders with climate change-related weather events. Multiple studies have shown that, after wildfires, depressive symptoms may be present in between one quarter and one third of participants [60]. Sullivan and colleagues [44] found that 23% of subjects presented with clinically significant depression post-disaster and new-onset depression was markedly more prevalent amongst those with pre-existing mental health problems versus those without (32.1% versus 12.6%, respectively). Depressive symptomatology rates are also reflected after hurricanes. In a study of adults affected by Hurricane Sandy, depressive symptoms were seen in 35.4% of subjects 12–28 months post-disaster [43]. Concerningly, one year after the initial evaluation, the rate of depressive symptoms was not significantly lower (30.8%, *p* = 0.39), which may reflect the longevity and pervasiveness of depression. A study on children in Grades 2 through 4 who lived through Hurricane Ike further supports this hypothesis–approximately 11% had depressive symptoms at both 8 and 15-months post-disaster, which underscores the continuity of depressive symptoms across time [41].

Chronic consequences of climate change may also have a significant impact on depression and other mood disorders. One study found significant associations between depression and high temperatures consistent with projected global warming increases [48]. Higher probabilities of self-reported anxiousness, hopelessness and meaninglessness have also been strongly linked to increases in temperature variability, another effect of climate change [58].

Proposed Mechanisms of Climate Change-Related Depression

The pathogenesis for depression is complicated, partially understood and multifactorial, including genetic, psychosocial and environmental factors [64]. The neurobiology of depression may be attributed to both structural and neurochemical changes in the brain, specifically as diminished prefrontal cortex regulation of the amygdala, hippocampus and hypothalamus coupled with dysregulation in the noradrenergic, cholinergic, dopaminergic and serotonergic pathways [64]. Studies have shown harmful environmental exposures from airborne pollutants such as diesel, carbon monoxide (CO), nitrogen oxides (NOx), sulfur dioxide (SO2), ozone (O3) and particulate matter can alter neurotransmitter function, both in the serotonergic and dopaminergic pathways [64–66], with implications for many psychiatric illnesses, including depression. Additionally, it is believed that exposure to stress, whether in acute climate change related events such as hurricanes or indirect effects from chronic climate change such as food insecurity or poverty, also increase the risk of depression [64]. Furthermore, climate change has direct neurobiological effects due to heat exposure and the subsequent increase in inflammation that is associated with greater likelihood of developing depression [64]. While there is a relatively large knowledge base examining climate change and the mechanisms by which cognition is affected, little research has been conducted examining the neurobiological implications climate change may have on depression. Future research should observe specific causal pathways of exposure to climate change-related events and neurobiological changes resulting in depression.

#### 3.5.3. Suicide

While less immediately catastrophic, minor changes to atmospheric composition can also have effects on mental health. Particulate matter such as ozone, sulfur dioxide and nitrogen oxide which are byproducts of climate change have been studied extensively. All have shown to be correlated with either a direct risk of suicide or with neuropsychiatric disorders that are associated with increased rates of suicidality, such as autism spectrum disorder, bipolar disorder, dementia and major depressive disorder [67–70]. The increased concentrations of these byproducts have been found under a variety of conditions, such as in those living near areas of high-traffic with several freeways, close to coal plants or oil refineries, or other sources of acute and chronic air pollution [68–70]. It is proposed that the pathophysiological mechanism behind air pollution and suicide is neuroinflammatory, as the air particulate matter acts as an irritant that generates local and systemic inflammatory responses, which can lead to increased rates of suicide, as well as vascular and pulmonary disorders [71,72].

Rising temperatures have also been independently linked to suicide, with spring and early summer having been identified as the period for greatest temperature-associated suicide risk [73,74]. A 1 °C increase in daily mean temperature was found to increase daily suicide risk by 1.4% in Republic of Korea [75]; in Taiwan, a 1 °C mean temperature increase led to a 1.8%, 2.34% and 1.45% increase in total, male and female suicide death numbers, respectively [76]. Studies have demonstrated that the relationship between temperature and suicide appears to remain stagnant, suggesting limited adaptation despite a consideration of factors including rising income, utilization and availability of air conditioning, or accessibility of mental health services [25,56]. One study also reported that economic factors such as unemployment and labor force participation were insignificantly related to completed suicides, suggesting a stronger role for climactic variables [76], while another study in India found the effect of temperature on annual suicide rates to be significant only during the growing season [54]. Similarly, droughts have been associated with increased suicide risk in farmers [73], as they may interfere with the livelihood of these people and thus increase psychological stress. Thus, while many physiological and behavioral mechanisms such as maladaptive serotonin receptors and disruption of sleep may be the primary basis for increased suicidality [56,77], it is integral not to undervalue the importance of economical mediators, particularly in vulnerable populations whose livelihoods are closely linked to changes in weather.

##### Proposed Mechanisms of Climate Change-Related Suicide

Several neurochemical endophenotypes including serotonin have long been known to be associated with emotion and behavior, including suicidal behavior. It is hypothesized that hopelessness, neuroticism, impulsivity and aggression have been associated with alterations in serotonin which are correlated with risk of completed suicide [78]. Both the noradrenergic and dopaminergic systems are maladaptive in depression, but the role of these in suicide is still unknown [66]. There is also a body of evidence that supports that stress and hypothalamic-pituitary-adrenal (HPA) axis dysregulation may play a large role in suicidal behavior [66]. Regarding climate change specifically, air pollution may cause hypoxia, which can dysregulate a variety of neurobiological systems, including the production of serotonin [64]. Studies have linked a correlation between hypoxia and suicide [78] and exposure to air pollutants (particulate matter, specifically) were consistently correlated with an increased prevalence of completed suicide [79]. This supports the hypothesis that exposure to air pollutants not only increases the risk of depression but also of completed suicides. These findings also provide evidence of possible neurobiological underpinnings.

### 3.6. Externalizing Behaviors

In addition to internalizing mental health outcomes, it is worth discussing externalizing behaviors associated with these conditions. Although less studied, externalizing behaviors, maladaptive actions that are directed toward the external environment, remain an important area of research, as changing climate can cause feelings of isolation and related stressors that can trigger aggressive behavior. Historically, the literature has focused more on how climate change affects collective unrest, aggression and violence amongst communities, rather than an individual’s externalizing behaviors. For instance, severe weather events (e.g., droughts) have been linked to societal conflicts and wars through stressors such as resource scarcities, forced displacements and loss of livelihood [80]. Even the chronic, more incremental changes of climate change are strongly linked to events of human conflict around the world: each standard deviation of change in climate towards higher temperatures or more extreme rainfall have been shown to increase frequency of intergroup conflict by 14% [81].

While the societal impacts of climate change on aggression are important, the focus of this review was on outcomes that reflected individual mental health outcomes. Like internalizing behavioral disorders, externalizing behavioral disorders occur significantly more in those exposed to acute climate-related disasters [82]. Studies directly measuring externalizing behavioral disorders were limited. Using violent crime as a proxy for individual aggression, one study found that over 16 year period, monthly mean temperature and violent crime rates were strongly correlated [57]. Measuring peripheral serotonin receptor densities in a small group of healthy males (N = 18, mean age 31.4 y) and male violent offenders (N = 33, mean age = 34.5 y), there also appeared to be a correlation between ambient temperature and peripheral serotonin receptor densities, which itself also correlated negatively with the incidence of violent crime. The authors thus proposed that effects of ambient temperature and individual violence may be mediated by a physiologic mechanism involving serotonergic pathways, also suggested elsewhere [83–85], and thus emphasizes the consideration of physiological effects on individual aggression in association with global climate change. Another study suggested a positive association between daily temperature and homicide rates in South Africa. A one-degree Celsius increase in same-day maximum temperature was associated with a 1.5% increase in homicides. South Africa has one of the highest homicide rates on record at an estimated 36 per 100,000 individuals, roughly six times the global average. South Africa is also experiencing climate warming at a faster rate than the global average, which heightens the importance of understanding potential relationships between temperature and adverse health and social outcomes such as homicide.

## 4. Discussion

The goal of this paper is to underscore the negative impacts climate change has had on mental health outcomes, particularly in vulnerable populations such as survivors of adverse weather events, those of lower socioeconomic status and those with pre-existing mental health conditions. Direct effects include increased frequency and/or severity of acute climate-related events such as hurricanes, floods and fires which may serve as exposures to developing disorders such as PTSD, depression and anxiety. Understanding the experience of the disaster and related stressors (e.g., fearing for one’s life, death of friend/family member), or the extent of subsequent life stressors may help identify those at higher risk for developing mental health consequences. An implication for healthcare providers and policymakers alike is to recognize that, while the rate of mental health problems after natural disasters may decrease over time, they may persist for many years and often occur at higher rates than at national levels [38].

In addition to the aspects discussed above, climate change can lead to other negative mental health outcomes that are not captured in this review but are important to note. For instance, case studies demonstrate the presence of an intense feeling of grief due to climate change-related loss, which can be directly observed from climate change-related disasters and their aftermaths, as well as from more gradual and ongoing ecological changes such those to as weather and landscape patterns. This phenomenon has been termed ‘ecological grief’ or ‘ecological anxiety’ [86]. Additionally, even concerns regarding climate change as an existential threat may detrimentally affect mental health outcomes [87, 88]. Further research should be done to give further validity to these experiences and investigate appropriate treatment modalities and implications at both the individual and macroscopic level.

Unsurprisingly, mental health service utilization increases during and succeeding extreme weather events [89] and providers and mental healthcare infrastructure must be properly prepared to meet the increased demand to adequately serve the needs of the population. Enhancing organizational supports by supplementing primary mental health services and identifying populations that may be particularly vulnerable to disasters and working prophylactically to enhance resiliency in these groups is paramount [90,91].

### 4.1. Interventions to Mitigate the Effect of Climate Change on Mental Health

This review underscores the necessity to further study and understand the link between climate change and mental health. Climate change is a serious challenge that poses substantial threats to life, health and wellbeing [92]. Unfortunately, those who are the most affected by disasters are those who can least afford to be affected by them. Understanding the persistent effects of weather-related events such as natural disasters and prolonged droughts, as well as the ability to identify vulnerable populations, can help communities and individuals adapt to these changes. Even relatively simple interventions, such as sharing stories and discussing mental health consequences amongst communities can greatly reduce depression [93] and improve a community’s capacity for adaptation following natural disasters. In vulnerable populations such as the elderly, where stoicism and maladaptive coping (e.g., venting/distraction) are common, there appears to be greater mental health deterioration after disasters [94]. In exchange, proactive approaches should be implemented to increase resilience and encourage protective coping strategies, such as socialization and communitarianism. On a larger scale, executing strategies and strengthening infrastructures in anticipation of threats from rising temperatures and droughts on agriculture, for instance, can decrease the individual’s burden. Therefore, it is implied that provision of subsidies and guaranteed income during the drought seasons may, for instance, lead to less economic and psychological stress on farmers and others who live and work in worrisome climates and thus positively impact mental health outcomes [15].

### 4.2. Areas for Future Research

Finally, continued research is of the utmost importance in gaining an even deeper understanding of the impact of climate change on mental health outcomes. As such, it is necessary to address limitations regarding research in this area. First, studies vary greatly in samples, methodologies, measures and outcomes, leading to findings that are often inconsistent and difficult to compare and thus limiting conclusions which can be made across studies. Second, most studies are limited by methodological weaknesses, such as small sample size, limited follow-up time (if any),the use of limited screening tools for mental health problems (sometimes only a few questions) or ED visits as a proxy for mental health, and few adequately controlled for important variables such as pre-existing mental health. Third, few studies examining the relationship between climate change and mental health have hypothesized and tested mediation and moderation effects of various environmental, behavioral and social factors, which will be salient when developing and implementing interventions to build resilience and increase adaptation. Ultimately, there is a strong bias in the existing literature towards internalizing behavioral outcomes, rather than externalizing behaviors and, while many historical studies observed relationships between increasing temperatures and societal aggression, recent studies on climate change effects and externalizing problems such as conduct disorder, for instance, are virtually non-existent. Future research should expand the scope to assess how the type, intensity, duration, and frequency of climate change events affect mental illness globally [4]. Future research should also strive for more rigorous methodologies and place a greater emphasis on longitudinal studies examining predictors as well as mediating and moderating relationships. Proper comprehension of these links is essential to better inform interventions in order to adapt to and mitigate climate-related mental health consequences.

## 5. Conclusions

After a thorough review of the literature, our study was able to draw three key conclusions: acute, subacute and chronic consequences of climate change are associated with a variety of negative sequelae in the context of internalizing and externalizing behaviors; these sequelae are likely due to multiple complex neurobiological mechanisms; and there are several implications to these changes, including increased stress on an already overtaxed mental health infrastructure, as well as detrimental socioeconomic impacts. These results have monumental implications, as climate change is expected to continue to degrade our planet and its inhabitants in the coming years and the ability to both adapt to and mitigate these effects will be of utmost importance for both physical and, as this review demonstrates, mental health. We recommend increased efforts in the creation and implementation of policies that combat the effects of climate changes, including mitigation of greenhouse gases, less reliance on fossil fuels, development of alternative energy sources, prevention of the loss of biodiversity and reduction in our individual carbon footprints. For this to occur, federal research funding that can target prevention and intervention strategies to mitigate climate change’s impact on mental health must be increased.

Climate change is one facet of a complex interconnection of environmental, physical, social and ecological aspects that affect mental health. However, as demonstrated in this paper, the significance of climate change and climate change-related weather events and their implications are immense. More research is needed to elucidate mediating, predictive and protective factors in the relationship of climate change and mental health. Concurrently, continued efforts to minimize the effects of climate change, such as mitigation of greenhouse gas emission and reducing our individual carbon footprints, are tangible steps. As the evidence for mental and behavioral outcomes associated with climate change increases, measures must be made to address both climate change and the responses to mental health effects. Climate change is projected to seriously impact our planet and its inhabitants in the coming years and the ability to both adapt to and mitigate these effects will be of utmost importance for both physical and, as this review demonstrates, mental health. Interdisciplinary researchers and healthcare professionals must advocate for research, education, and policies that support disaster-resilient infrastructure and human services that can help communities across the globe mitigate the effects of climate change [4]. In conclusion, it is of the utmost importance that we implement all the measures we can to combat anthropogenic climate change in order to limit its effects on mental health.

## Figures and Tables

**Table 1.** Recent studies categorized by type of climate change-related event.

Exposure Category	Study	Sample * N (% Female) Age	Study Location	Study Design and Period	Exposure (s) of Interest	Mental Health Outcome: Measure(s)	Major Findings
Acute							
Dust storm	Lee et al. (2019) [34]	N = 30,704 (33.8%)	Republic of Korea: Seoul	Ecological 2002–2015	Dust storms Duration and intensity	Completed suicide	Suicide counts during Asian dust storm (ADS) days were significantly higher than those during non-dust storm days. Exposure to ADS was associated with a 13.1% (95% CI, 4.5–22.4, p = 0.02) increase in suicide risk on an ADS day. For every day increase in duration, an additional 7.7% (95% CI, 2.9–12.8, p = 0.02) risk in completed suicide.
Flood	Mulchandani et al. (2020) [35]	N = 819	UK: England	Longitudinal 2015–2018	Flooding Exposure and experience	Depression: PHQ-2 Anxiety; GAD-2 PTSD: PCL-6	Approximately 5.7% of participants reported symptoms of probable depression, 8.1% probable anxiety and 11.8% probable PTSD, with prevalence of all mental disorders higher in the flooded versus unaffected group. 9.4%of participants reported persistent damage to their homes. A significant reduction in prevalence for all probable mental health diagnoses was observed in flooded group over three years.
	Thomas et al. (2021) [36]	N = 171 (69%) Age: 18–65 y	India: Karnataka	Cross-sectional December 2018-January 2019	Flooding Exposure and experience	Presence of psychiatric symptoms (depression, anxiety, somatic disorders, sleep problems and substance misuse)	After experiencing a flood, 66.7% of the sample showed the presence of symptoms consistent with at least one psychiatric problem. This, however, was not statistically significant. Depression and anxiety were the most prevalent symptoms, 33% and 31%, respectively. A statistically significant correlation with property damage or destruction of houses and psychiatric symptoms was observed.
Fire	Bryant et al. (2014) [37]	N = 1017 (60.3%) Age: ≥18 y at time of fire (M = 53.1 y)	Australia: Victoria	Cross-sectional 2011–2013	Victorian Black Saturday Bushfires (February 2009–March 2009) Exposure and experience	Depression: PHQ-9 PTSD: PCL Non-specific psychological distress: K6	Rates of any disorder significantly differed in high-, medium- and low-affected areas at 43.1%. 33.3%, 27.5%, respectively (*p* < 0.001). Probable PTSD and depression in 15.6% and 12.9% of subjects in high-affected areas, respectively. Both significantly more than medium- and low-affected areas (*p* < 0.01) Death of someone, fear for one’s life and subsequent major life stressors were predictors for PTSD, depression and serious mental illness. Additional predictors for fire-related PTSD included female sex and lower educational level.
	Bryant et al. (2018) [38]	N = 735 (61.5%) Age: ≥18 y at time of fire (M = 53.5 y)	Australia: Victoria	Longitudinal 2011–2014 T1: December 2011–January 2013 T2: July 2014-November 2014 (5 y PD)	Victorian Black Saturday Bushfires (February 2009–March 2009) Exposure and experience	Depression: PHQ-9 PTSD: PCL Non-specific psychological distress: K6	At 5 years post-disaster, rates of symptoms decreased for PTSD (8.7% vs. 12.1%), depression (9.0% vs. 10.9%) and serious mental illness (5.4% vs. 7.8%) but remained higher than national levels. Extent of recent life stressors was the most robust predictor of later development of firerelated PTSD (OR 2.11; 95% CI [1.22, 3.65]) and depression (OR 2.86; 95% CI [1.74, 4.70]).
Hurricane	Bozick (2021) [39]	N = 5694 (57.4%) Age: ≥18	USA: Texas	Observational w/a probability sample 2017–2018 T1: Pre-Harvey T2: Post-Harvey	Hurricane Harvey (August 2017) Exposure and experience	Number of days in the past 30 days with poor mental health	Statistically significant differences between the pre- and post-Harvey samples in both poor physical and poor mental health. Rate of experiencing days with poor mental health is 32% higher in post-Harvey sample compared with the pre-Harvey sample (95% CI, p < 0.01). Following the storm, adults experienced an increase of 1.31 days a month of poor mental health (5.38 days total), equating to an additional 12,371 person-years of poor mental health in the span of one month.
	Garfin (2022) [40]	T1: N = 1637 (54.6%) Age: ≥18	USA: Florida	Longitudinal 2017–2018 T1: Pre-Irma T2: 1 month PD T3: Post-Michael	Hurricane Irma (September 2017); Hurricane Michael (October 2018 - November 2018) Effects of repeated exposures	Posttraumatic stress symptoms (PTSS) Global distress: BSI-18	Prior mental health conditions (b, 0.18; 95% CI, 0.07–0.28), prior hurricane-related loss and/or injury (b, 0.09; 95% CI, 0.02–0.17), hours of Irma-related media exposure (b, 0.03; 95% CI, 0.02–0.04), being in an evacuation zone and not evacuating (b, 0.14; 95% CI, 0.02–0.27) and loss and/or injury after Hurricane Irma (b, 0.35; 95% CI, 0.25–0.44) were positively associated with post-traumatic stress symptoms after Hurricane Irma. Similar results were observed with Hurricane Michael-related exposures. After Hurricane Michael, prior mental health ailments, posttraumatic stress symptoms related to hurricanes Irma and Michael were associated with overall functional impairment.
	Lai et al. (2013) [41]	N = 277 (52%) Age: Grades 2–4 (M = 8.7 y)	USA: Texas	Longitudinal May 2009–December 2009 T1: 8 months PD T2: 15 months PD	Hurricane Ike (September 2008) Exposure and experience	Depression: CDI PTSD: PTSD-RI-R	At 8 months post-disaster, 23% of children had clinically significant PTSD symptoms, 43% had clinically significant depressive symptoms and 10% had comorbid PTSD and depressive symptoms At 15 months post disaster, these decreased to 14%, 18% and 7% respectively. 33% of those with comorbid symptoms at 8 months continued to have comorbid symptoms at 15 months. Children with comorbid symptoms had poorer recovery, more severe symptoms and reported greater exposure and recovery stressors
	Orengo-Aguayo et al. (2019) [42]	N = 96,108 (50.3%) Age: Grades 3–12	Puerto Rico	Cross-sectional February 2018–June 2018 (5–9 months PD)	Hurricane Maria (September 2017) Exposure to hurricane-related stressors	Depression: NCTSN-HART PTSD: NCTSN-HART	Clinically significant symptoms of PTSD in 7.2% of children; more in girls (8.2%) than boys (6.1%; *p* < 0.001). Girls also displayed higher depression scores than boys (*p* < 0.001). Approximately 30% of subjects perceived their lives to be at risk during the disaster, with girls experiencing this more than boys (34.2% vs. 27.3%; *p* < 0.001). Exposure to hurricane-related stressors moderately correlated with PTSD symptoms. Ongoing loss and disruption, geographical location and SES were not.
	Schwartz et al. (2017) [43]	N = 130 (77.7%) Age: 18–92 y (M = 49.73 y)	USA: New York	Longitudinal T1: 11–28 months PD (M = 14.5 months) T2: 1 y after T1	Hurricane Sandy (October 2012–November 2012) Personal and/or property exposures	Anxiety: PHQ-4 Depression: PHQ-4 PTSD: PCL-S	Overall, symptom prevalence decreased from T1 to T2. This was significant for anxiety (50.0% to 41.5%; *p* < 0.01) and PTSD (29.2% to 24.8%; *p* = 0.001) but not depression (35.4% to 30.8%; *p* < 0.39). At T2, experiencing personal damage, property damage, or both was positively associated with PTSD symptoms, but not anxiety or depression symptoms. Strongest predictor of experiencing a mental health symptom at T2 was having symptoms at T1.
	Sullivan et al. (2013) [44]	N = 498 (0%) Male veterans only Age: 18–60 y	USA: Louisiana	Cross-sectional 2007–2008 (2.5 y PD)	Hurricane Katrina (August 2005) Stressful hurricane-related events	Depression: PHQ-2 Anxiety: GAD-7 Panic disorder: PHQ PTSD: SPRINT	Compared to veterans without (N = 250) preexisting mental disorders, those with preexisting disorders (N = 248) were almost 7 times more likely to screen positive for any new mental disorder. Preexisting PTSD had the highest risk for new mental illness, followed by schizophrenia then affective disorders Of veterans with preexisting mental disorders, 72% screened positive for new mental disorder. Almost a third of those without preexisting mental illness screened positive for at least one of mental disorder.
Multiple Exposures	Edwards et al. (2021) [45]	N = 4592 (unknown) Age: 10 y	Philippines	Longitudinal 2016–2017 (15 y PD)	Cumulative disaster exposure (tropical cyclones, extreme rainfall, drought, volcanic activity, storm surges, sea level rises, flooding, tsunamis, earthquakes, fire, marine pollution) Hazard occurred in last 3 years. (Y/N)	Food insecurity: FIES; Stunting: WHO Child Growth Standards; Caregiver stress: PSS; Caregiver depression CES-D; Family violence, witnessing violence, child physical abuse (Y/N)	On average, children were exposed to 2.87 disasters from 2014 to 2017. 41% of children experienced family violence and 33% of children reported witnessing violence in the past 12 months. 30% of children reported being physically hurt by a parent, with 20% being hurt by parents in a forceful manner. 35% of children were stunted and 35% of caregivers report severe food insecurity. Exposure to a greater number of disasters were associated with an increased chance of children witnessing violence, being hurt by an adult, hurt by a parent and hurt by a parent forcefully.
	Graham et al. (2019) [46]	N = 7525 (59.3%) Age: ≥16 y	UK	Cross-sectional 2013–2015	Severe weather (wind, rain, snow, flooding) Home damaged by severe weather event in last 6 months? (Y/N)	Common mental disorder: CIS-R PTSD: PLC-C Suicidal thoughts, non-fatal suicide attempts and self-harm by questionnaire	Subjects who lived in storm or flood-damaged homes in 6 months prior to interview had significantly increased risk of having a common mental disorder (*p* < 0.01), suicidal ideation (*p* < 0.01) and ever having attempted suicide (*p* < 0.01). Associations with PTSD (*p* = 0.06) and with recent use of mental health treatment/services was not significant (*p* = 0.06)
Subacute							
Drought	Luong et al. (2021) [47]	Baseline: N = 2607 (59%) Pooled cross-sectional: N = 6519 (61%) Age: ≥18 y	Australia: New. South Wales	Ecological/longitudinal 2007–2013	Drought HDSI (measures agricultural drought); SPEI (measures meteorological drought)	Psychological distress: K10	Significant non-linear relationship between drought exposure and psychological distress (HDSI; *p* < 0.001; SPEI; *p* < 0.001). Results show an inverted Ushape, where drought initially correlates with increased psychological distress and the turning point (where distress begins to decrease) occurs around 3 years after drought.
Heat	Lee et al. (2018) [48]	N = 166,579 ED admissions for mental disease Age: –	Republic of Korea	Ecological 2003–2013	Heatwaves Temp (daily mean), humidity, total solar radiation	ED admissions related to mental health	Strongest association between mental health diagnoses (specifically anxiety, schizophrenia, dementia and depression) and high temp occurred within 0–4 days of high temp exposure. 14.6% of emergency admissions for mental disease were attributable to extreme hot temperatures, with anxiety the highest risk (31.6%) and elderly more susceptible (19.1%)
	Dang et al. (2022) [49]	N = 7780 Age: −18 y	Vietnam: Ho Chi Minh	Cross-sectional 2017–2019	Heatwaves Combination of intensity (≥97th percentile of the daily mean temp, 30.9 °C) and duration (≥2 continuous days)	Admissions for psychiatric illness to Ho Chi Minh Mental Health Hospital	Heatwaves increased allcause psychiatric hospitalization by 62% (95% CI, 36–93%) for the main effect and by 8% (95% CI, −3 to 19%) for added effect of heatwaves. The group age 18–60 was primarily affected by the main effect of the heatwave, while the group aged 61 and older was affected by added effects of the heatwave. Hospitalizations due to psychoactive substance use was significantly affected by the main effect of heatwaves (RR: 2.21; 95%CI:1.55–3.15) and psychotic disorders were highly vulnerable to the main and added effects of heatwaves with RR = 1.50 (95% CI, 1.20–1.86) and RR = 1.14 (95% CI, 1.01–1.30), respectively.
	Ngu et al. (2021) [50]	–	60 countries worldwide did	Ecological	Heatwaves Temperature and/or relative humidity	Completed suicide	In countries with a significant result, an increase in suicide of 3.5% is observed for every unit increase of heatwave counts. Around half the countries showed a significant increase in suicide with respect to relative humidity, with the per unit increase of relative humidity ranging from −6 to +4.9%.
	Yoo et al. (2021) [51]	N = 2.8 million (39.4%)	USA: New York State	Ecological 2009–2016	Heatwaves (27.07 °C and above)	ED visits for specific mental disorders	Acute exposure to high heat (27.07 °C and above) increased ED visits for mental disorders (substance use, mood and anxiety disorders, schizophrenia and dementia). No statistically significant difference in subgroups in the sample being more vulnerable to the effects of heatwaves.
Chronic							
	Basu et al. (2018) [52]	N = 219,942 ED visits (44%)	USA: California	Ecological 2005–2013	Temp (daily mean, maximum, minimum)	ED visits related to mental health, external-cause injuries	During the warm season, a 5.6 °C (10 °F) increase in same-day mean apparent temperature was associated with 4.8%, 5.8% and 7.9% increases in the risk of visits for mental health disorders, self-injury/suicide and intentional injury/homicide, respectively. Greatest risk: Hispanics, whites, persons aged 6–18 years and females
	Bundo et al. (2021)[53]	N = 89,996 (50.1%) Age: ≥18 y	Switzerland: Bern	Ecological 1973–2017	Temp daily mean	Psychiatric hospitalizations	For every 10 °C increase in daily mean temperature, the risk of hospitalization due to mental disorders increased linearly by 4.0% (RR: 1.04; 95% CI 1.02, 1.07), even while controlling for air pollution and other meteorological factors. Greatest risk: diagnoses of schizophrenia or developmental disorders,
	Burke et al. (2018) [25]	N(USA) = 851,088 N(Mexico) = 611, 366 Age: –	USA, Mexico	Ecological US: 1968–2004 Mexico: 1990–2010	Temp monthly mean, monthly precipitation data	Completed suicide	For a 1 °C rise in monthly mean temp, 0.6% increase in suicide rates in US counties and 2.1% in Mexican municipalities. Effect similar in hotter vs. cooler regions. Unlike all-cause mortality, suicide increased at hotter temperatures, decreased at colder temperatures and the effect has not decreased over time or with increasing income or the adoption of air conditioning.
	Carleton et al. (2017) [54]	-	India	Ecological 1956–2000	Temp daily mean	Completed suicide	For days above 20 °C, a 1 °C increase in a single day’s temp during the growing season increased annual suicides by 0.008 per 100,000 people. Significant effect only seen during India’s growing season; no identifiable impact in non-growing season.
	Middleton et al. (2021) [55]	N = 5373 (unknown) Age: –	Canada: Newfoundland and Labrador	Ecological 2012–2018	Temp Critical threshold range; daily mean temperature	Mental health-related visits, including suicide related-visits	Daily mean temperature ranged between −31.4 °C to 26.8 °C and critical threshold range identified as −5 °C to 5 °C, with 17 days as the longest consecutive number of CTR days in a row. Incidence of mental health-related visits was significantly higher when the cumulative average of daily temperatures two weeks prior was warmer (above −5 °C) compared to cold temperatures (less than −5 °C).
	Mullins et al. (2019) [56]	N(ED) = 8294 N(Suicides) = 2,096,460 N(Self-reported mental health) = 4,120,514	USA: California	Ecological ED visits: 2005–2016 Suicide: 1960–2016 Self-reported mental health: 1993–2012	Temp daily mean, daily precipitation, humidity, daily sunlight	ED visits related to mental health Self-reported mental health status: BFRSS Suicide rates	Higher temp increased ED visits for mental illness, suicides and self-reported days of poor mental health No evidence of adaption: temp relationship stable across time and despite factors such as baseline climate, air conditioning penetration rates and accessibility of mental health services
	Page et al. (2018) [5]	N = 22,562 Age: ≥65; ≤65	United Kingdom	Cross-sectional 1998–2007	Temp daily mean	Mean daily count of deaths Diagnosis of psychosis, dementia or substance use	Individuals with a diagnosis of psychosis, dementia, or substance misuse had a 4.9% higher risk of death (95% CI [2.0–7.8]) per 1 °C increase in temperature above the 93rd percentile of the annual temperature. Younger individuals with a primary diagnosis of substance use disorder displayed the highest mortality risk.
	Tlihonen et al. (2017) [57]	N = 551,529 violent crimes	Finland	Ecological 1996–2013	Temp Monthly mean	Violent Crime (proxy for aggression)	A strong correlation was observed between the monthly violent crime rate and monthly mean ambient T (r = 0.51, *p* < 0.001). Ambient temp explained 10% of variance in violent crime, with 1.7% increase per 1 °C.
		N = 51 (0%) Males only - n(violent) = 33 - n(healthy) = 18 Age: M = 33.4 y	Finland	Cross-sectional 1996–1997	Temp Monthly mean	Peripheral serotonin transporter density	Among healthy group, temp correlated significantly with peripheral serotonin transporter density (r = −0.64, *p* = 0.0025), which itself correlated negatively with the incidence of violent crime (r = −0.56, *p* = 0.06). This was even stronger among violent offenders (r = −0.68, *p* = 0.02.), suggesting mediation by serotonergic system. No offenders suffered a DSM axis-1 mental disorder aside from substance use and none were on any serotonin-altering drugs
	Vida et al. (2012) [16]	N = 347,552 ED visits related to mental health Age: ≥15 y	Canada: Québec	Ecological 1995–2007	Temp daily mean, relative humidity	ED visits related to mental health	ED visits increased with increasing mean temp, with effects most notable in metropolitan and suburban areas. Incidence ratio risks increased with increased temp. At 22.5 °C (72.5 °F) and 25 °C (77.0 °F), the number was usually significantly higher than average. Visits increased with humidity in the younger age group.
	Xue et al. (2019) [58]	N = 21,543 adults Age: –	China	Difference-in-difference study 2010–2014	Temp Long-term level of temp, temp variability	Self-reported mental health scores: Center for Epidemiologic Studies Depression Scale test	A 1 °C increase in temp variability (e.g., SD of daily temp within a calendar year) was correlated with 15% risk of decreased mental health score. Effects were strongly linked to a higher probability of feeling nervous, upset, hopelessness and meaninglessness. No significant association between annual temperature mean and mental health score.

* % Female and age data noted if available for sample population. Abbreviations: CI = confidence interval; ED = emergency department; M = mean; OR = odds ratio; PD = Post-disaster; PTSD = Post-traumatic stress disorder; RR = relative risk; SES = socioeconomic status; SD = standard deviation; UK = United Kingdom; US = United States of America. Abbreviations-Measures: AAQ = Anger Attacks Questionnaire; BRFSS = Behavioral Risk Factor Surveillance System; BSI-18 = Brief Symptom Inventory-18; CES-D = Centre for Epidemiological Studies Depression Scale; CDI = Children’s Depression Inventory; CIS-R = Clinical Interview Schedule-Revised; DISC-IV = Diagnostic Interview Schedule for Children IV; FIES = Food Insecurity Experience Scale; GAD-7 = Generalized Anxiety Disorder, 7 item; GAI = Geriatric Anxiety Inventory; HDSI = Hutchinson Drought Severity Index; ICD = International Classification of Diseases; IES-R = Impact of Scale-Revised; K10 = Kessler Psychological Distress Scale, 10 item; K6 = Kessler Psychological Distress Scale, 6 item; NCTSN-HART = National Child Traumatic Stress Network Hurricane Assessment and Referral Tool; PCL = PTSD Checklist; PCL-S = PTSD Checklist-Specific; PHQ = Patient Health Questionnaire; PSS = Perceived Stress Scale; PTSD-RI-R = Post-traumatic Stress Disorder-Reaction Index, Revision 1; PTSS = Post-traumatic Stress Symptoms; SPEI = Standardized Precipitation Evapotranspiration Index; SPRINT = Short PTSD Rating Interview.

## Data Availability

This is a review article. All data were gathered from the available literature.
